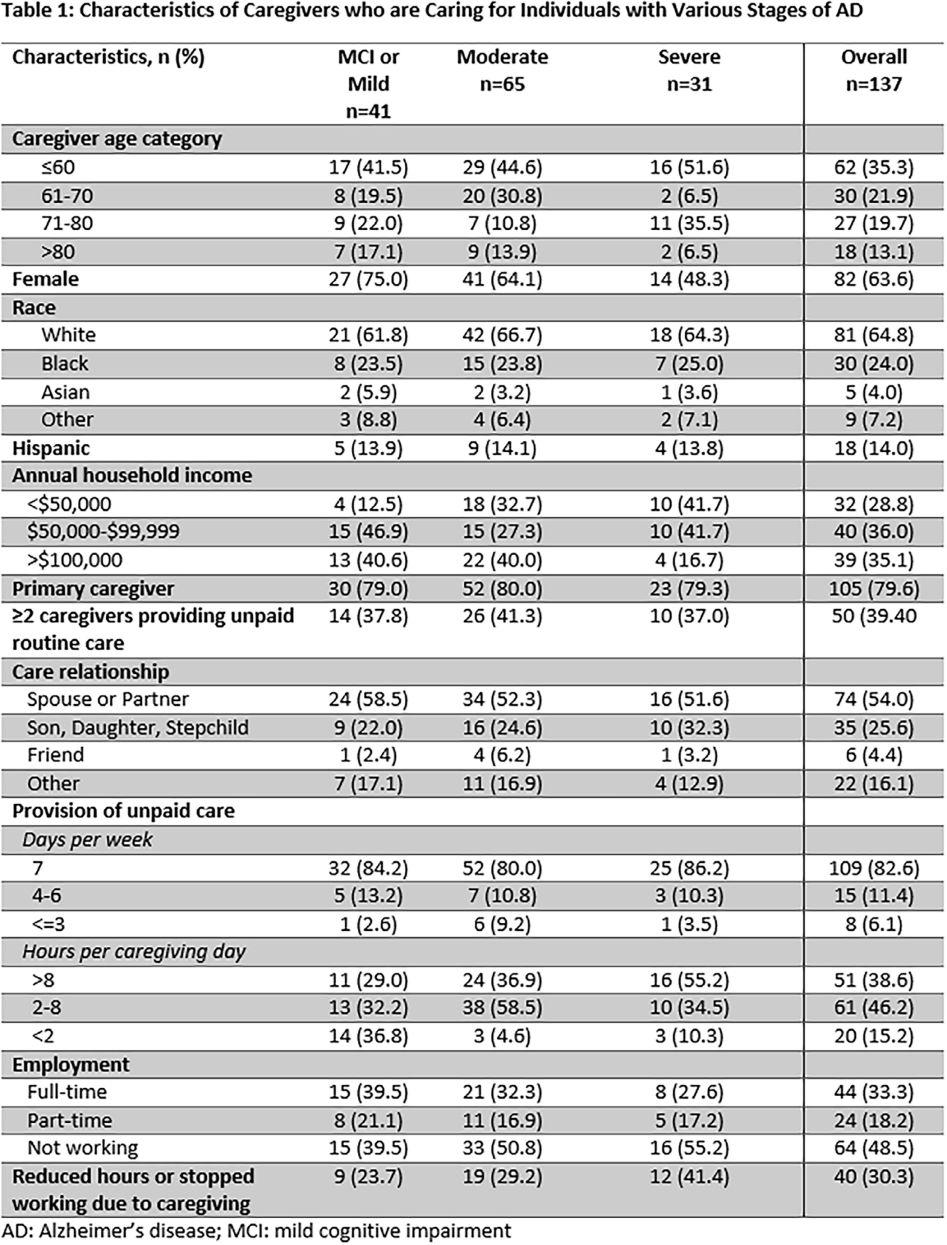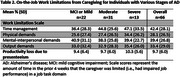# Employment Outcomes and Productivity Loss Among Caregivers of Community‐Dwelling Individuals with Alzheimer's Disease

**DOI:** 10.1002/alz70860_101255

**Published:** 2025-12-23

**Authors:** Patricia G Synnott, Joshua T Cohen, Theresa Frangiosa, Amber Roniger, Peter J Neumann, Tara Lavelle, Pei‐Jung Lin

**Affiliations:** ^1^ Tufts Medical Center, Boston, MA, USA; ^2^ UsAgainstAlzheimer's, Washington, DC, USA

## Abstract

**Background:**

As a person's Alzheimer's disease (AD) progresses, caregivers can experience increasing emotional, physical, and financial demands that impair their work productivity. Understanding how caring for individuals with AD affects caregiver employment and work performance as the disease advances is critical for informing support services and policies to address caregiver needs. We examined employment and productivity loss among caregivers of community‐dwelling individuals with AD.

**Method:**

We conducted a web‐based survey of U.S. adults providing unpaid care to individuals with AD. The survey was sent to 6,037 individuals affiliated with UsAgainstAlzheimer's and other research and advocacy organizations. Caregiver respondents reported the care recipient's AD stage (mild cognitive impairment [MCI], mild, moderate, and severe) based on symptom descriptions. We used the Short‐Form Caregiver Work Limitations Questionnaire to measure work limitations in time management, physical demands, mental‐interpersonal demands, and output, and to estimate productivity loss due to presenteeism (i.e., reduced productivity while working). Analyses were stratified by the care recipient's AD severity.

**Result:**

Our analytic sample comprised 137 individuals, of whom 41 cared for individuals with MCI or mild dementia, 65 with moderate, and 31 with severe dementia (Table 1). Full‐time employment was more common among caregivers of individuals with milder AD stages. A greater proportion of caregivers of individuals with severe dementia (41%) reported reducing work hours or leaving the workforce due to caregiving responsibilities, compared to caregivers for MCI/mild (24%) and moderate AD (29%), although differences did not reach statistical significance.

Employed caregivers (*n* = 66) reported that caregiving made it difficult to meet work time demands 42% of the time, with greater impairments among caregivers of individuals with severe dementia (Table 2). Productivity loss due to presenteeism was approximately 10% across all AD stages, considerably exceeding the 2.7% average for the general U.S. population.

**Conclusion:**

Caregivers may face increasing difficulty maintaining employment as their care recipient's AD progresses. Among those who remain employed, productivity losses due to presenteeism persist at elevated levels across all disease stages. These findings highlight the need for policies, such as flexible work arrangements and caregiving support systems, to address employment challenges and productivity losses experienced by caregivers of individuals with AD.